# Metformin Ameliorates Gestational Diabetes Mellitus-Induced Endothelial Dysfunction *via* Downregulation of p65 and Upregulation of Nrf2

**DOI:** 10.3389/fphar.2020.575390

**Published:** 2020-10-09

**Authors:** Cong Cong Sun, Ya Nan Lai, Wen Huan Wang, Xiao Min Xu, Xiao Qing Li, Hai Wang, Jia Yong Zheng, Jian Qiong Zheng

**Affiliations:** ^1^ Wenzhou Key Laboratory of Obstetrics and Gynecology, The Third Affiliated Clinical Institute of Wenzhou Medical University, Wenzhou People’s Hospital, Wenzhou Maternal and Child Health Care Hospital, Wenzhou, China; ^2^ Department of Obstetrics and Gynecology, The Third Affiliated Clinical Institute of Wenzhou Medical University, Wenzhou People’s Hospital, Wenzhou Maternal and Child Health Care Hospital, Wenzhou, China

**Keywords:** gestational diabetes mellitus, metformin, endothelial dysfunction, p65, Nrf2

## Abstract

Gestational diabetes mellitus (GDM) causes oxidative stress in mothers and infants and causes vascular endothelial dysfunction, which is a key factor for maternal and fetal cardiovascular diseases in the later stage of GDM, seriously threatening the life and health of mothers and infants. Nowadays, metformin (MET) has been discovered to improve endothelial function, but studies regarding the mechanism of MET improving endothelial cell function and alleviating endothelial function under hyperglycemia are still extremely limited. We aimed to investigate whether MET exerts its protective role against hyperglycemia-induced endothelial dysfunction through p65 and Nrf2. In our studies, applying cell migration assay and tube formation assay, we observed an obvious improvement of endothelial function under MET-treated, as characterized by that MET accelerated GDM-attenuated migration and angiogenesis of HUVECs. And ELISA assay results uncovered that Nrf2 expression level was decreased in GDM placenta, HVUECs and maternal serum comparing with normal group, however activation Nrf2 largely ameliorated tube formation under hyperglycemic condition. Furthermore, MET elevated the Nrf2 expression level and the level of nuclear Nrf2 accumulation in hyperglycemic HUVECs. Besides, preliminary evidence predicted that Nrf2 expression was modulated by transcription factor p65, which was increased in GDM peripheral blood, placenta and HUVECs, and suppression of p65 could recover GDM-induced suppression of angiogenesis. In addition, we also confirmed MET restores the GDM-induced angiogenesis impairment may *via* downregulation of p65 and upregulation of Nrf2. Taken together, the endothelial protective effect of MET under GDM (HG) conditions could be partly attributed to its role in downregulating p65 and upregulating Nrf2.

## Introduction

Gestational diabetes mellitus (GDM) is defined as glucose intolerance with onset or first recognition during pregnancy (Pons et al., 2015). GDM influences about 3%–25% pregnancies worldwide and the increasing incidence of GDM is mainly due to the increasing prevalence of short- and long-term complications and adverse pregnancy outcomes in GDM mother and offspring, such as type 2 diabetes mellitus (T2DM) or other metabolic disorders (Thorpe et al., 2005; Moyer VA, 2014; Jovanovič et al., 2015). GDM is considered a prediabetic state, while it shares a series of typical pathophysiological characteristics with type 2 diabetes including insulin resistance, hyperglycemia, biochemical abnormalities, and hyperlipidemia (Yefet et al., 2019). A great number of reports have revealed that the microvascular cell and vascular endothelial cell (VECs) dysfunctions occurred in the umbilical cord and placenta in GDM might lead to immediate or long-term cardiovascular disease risk (Di Fulvio et al., 2014; Sultan et al., 2015; Zhou et al., 2016; Amrithraj et al., 2017). And endothelial dysfunction is a key cause of pathogenesis when pregnancy metabolic disorders occur, including GDM, hypertensive disorders, obesity, and hyperlipidemia (Echeverria et al., 2020). Further, endothelial dysfunction could occur before pregnancy and predisposes women to hypertension in pregnancy and pre-eclampsia, and the latter could induce long-term changes in endothelial function, which could have implications for development of cardiovascular disease in later life (Karthikeyan and Lip, 2011). Vascular endothelial dysfunction, as characterized by the turbulence in VECs proliferation, migration, and angiogenesis led by GDM, is linked through the inflammatory response and oxidative stress (Desoye, 2018; Pardo et al., 2018). Up till now, the mechanism of how the dysfunction and injury of VECs occurred in GDM patients still remains unclear, and there is a lack of data concerning the intervening measures for protecting GDM women and offspring against cardiovascular risk.

The role of inflammatory response and oxidative stress in modulating diverse physiological functions, including reverting endothelial dysfunction induced by GDM has drawn more and more interest in recent years. Basically, inflammatory response and oxidative stress have been associated with altered expression levels of various factors. Nuclear factor erythroid 2-related factor 2 (Nrf2) has been implicated to play crucial roles in the control of inflammation, and is a master modulator of redox homeostasis *via* accommodation action among a battery of cytoprotective genes (Innamorato et al., 2009; Jazwa and Cuadrado, 2010; Bryan et al., 2013) to protect against diabetes-induced endothelial dysfunction (Sharma et al., 2017). Metformin (1, 1-dimethylbiguanide hydrochloride, MET) has been demonstrated to exert anti-oxidative and anti-inflammatory activities *via* activating the Nrf2 pathway in diabetes-associated macrovascular and renal injury (Wu et al., 2016). Thus, the potential roles of Nrf2 under the GDM condition with MET treatment has drawn our interest.

MET, an oral hypoglycemic agent, has been widely applied as a standard first-line therapy for T2DM due to its low cost, safety profile, and potential cardiovascular benefits (Zhou et al., 2001; DeFronzo et al., 2016), which has been shown to exert hypoglycemic effect by suppressing hepatic gluconeogenesis caused by elevated secretion of GLP-1 and related peptides *via* an intestinal AMP-activated protein kinase-dependent pathway (Inzucchi et al., 2014). MET has also been identified to have the function of preventing excessive gestational weight gain and therefore could be used to prevent excessive weight gain during pregnancy (Balsells et al., 2015; Bettencourt-Silva et al., 2019). Thus, MET is now the preferred option for GDM treatment considering its advantage of reducing maternal weight gain compared with insulin. In addition, if MET is used starting before pregnancy and until the end of the term in women with polycystic ovary syndrome, it can benefit both the mother (reducing risk of GDM, gestational hypertension, and preterm labor) and the foetus development (reducing risk of early pregnancy loss and foetal growth retardation) (Hyer et al., 2018). And increasing evidence has highlighted that MET can improve endothelial function, prevent vascular abnormalities caused by hyperglycemia and enhance the neovascularization of endothelial progenitor cells (EPCs) (Mather et al., 2001; Desouza, 2013). Whether the favorable functions of MET are related to its efficacy on blood glucose control is still unclear and remains paradoxical. Thus, the impact of MET on GDM needs further clariﬁcation because as previously reported, MET treatment provides potential protective effect on human umbilical vein endothelial cell (HUVECs) under high glucose condition.

Our study aimed to investigate how VEC dysfunction occurs under high-glucose (HG) or GDM conditions, which may provide a theoretical basis and ideal therapeutic target for preventing GDM individuals from cardiovascular complications and improve pregnancy outcomes. Here we provide evidence that MET ameliorates GDM-triggered endothelial dysfunction by enhancing Nrf2 expression level, which sheds new light on MET regulation of GDM-impaired endothelial function.

## Material and Methods

### Subjects

This study was approved by the Ethics Committee of the Wenzhou people’s hospital. All the individuals enrolled in this study provided voluntary informed consent and then accepted an oral glucose tolerance test (OGTT) at 24–28 weeks of gestation. All the control subjects (Normal) had a negative OGTT result, and GDM was diagnosed based on the WHO/IADPSG criteria: either a fasting plasma glucose level of ≧̸ 5.6 mmol/L or a 2-h postload plasma glucose level of ≧̸ 7.8 mmol/L (Pons et al., 2015), while without other pregnancy complications, including chronic gastrointestinal diseases; pre-pregnancy diabetes; celiac disease; a history of eating disorders, such as anorexia or bulimia; vegan, vegetarian, or macrobiotic regimens; and non-Caucasian ethnicity. Pregnant women with asthma, smokers and pregnancies with other maternal or foetal conditions (hypertension, preeclampsia, and placenta praevia) were also excluded. The diet and lifestyle of all the GDM subjects during the rest time of pregnancy were managed and modified. The clinical characteristics of the subjects are shown in **Table 1**.

**Table 1:** Clinical characteristics of normal and gestational diabetes mellitus (GDM) pregnancies.

Subjects	Normal	GDM	P
N	40	51	
Maternal BMI, kg/m^2^	25 (22–29)	30.2 (28.1–32.8)	0.032
Age, y	28 (23–36)	31 (20–43)	0.029
Gestational age at delivery, wks	39.5 (38–40.1)	38.6 (37.5–39.4)	0.072
Fasting glucose, mmol/L	4.4 (3.8–6.0)	5.1(3.8–8.7)	0.045
1-h glucose, mmol/L	7.2 (4.8–9.7)	11.5 (10.1–16.2)	0.002
2-h glucose, mmol/L	6.6 (3.9–8.8)	9.5 (6.1–13.2)	0.005

n. Normally distributed data are presented as means (min-max).

Placentae were taken from full-term (>37 weeks) pregnancies upon cesarean section. No significant difference was found in placental weight or birth weight between the two study groups. About one cubic centimetre of placental tissue was cut from the foetal surface around the umbilical cord of the placenta, cut into small pieces on ice, ground into powder in liquid nitrogen, and then put in lysis buffer and shaken at 4°C for 4 h. After a centrifugation at 15,000 rpm for 15 min, the supernatant was used for Western blot analysis.

Anthropometric measures and fasting blood sample collection (for detection of Nrf2 and p65 levels) were performed during the recruitment visit three days after GDM diagnosis. Maternal venous blood was obtained from peripheral venipuncture, and after centrifugation, the serum was harvested and stored at -80°C. Detections of OGTT, p65, and Nrf2 were all performed in the same analysis laboratory (Wenzhou Key Laboratory of Obstetrics and Gynecology, Zhejiang, China). All the women whose OGTT levels were positive following a diagnosis of GDM were invited to participate in this study.

### Cell Culture

A human umbilical vein fusion cell line EA.hy926 was purchased from American Type Cell Collection (ATCC), and maintained in high glucose Dulbecco’s Modified Eagle’s medium (DMEM, Gibco, 11054020) supplemented with 10% fetal bovine serum (FBS, Gibco, 10100147) and 1% penicillin-streptomycin (Gibco, 15070063).

HUVECs were extracted, isolated and cultured as described previously (Amrithraj et al., 2017). Umbilical cords collected from 12 GDM women (GDM was diagnosed at 24-28th gestational week) and from 10 normal women were subjected to digestion, the suspension was centrifuged (200 g, 5 min), and the HUVECs were harvested and cultured in EGM™-2 Microvascular medium (Lonza, CC-3156 & CC-4147) for rapid proliferation or in EBM™-2 Basal Medium (Lonza, CC-3156) supplemented with EGM™-2 SingleQuots™ Supplements (Lonza, CC-4176) for maintaining culture. The primary HUVECs split for less than 5-7 passages were used for experiments.

### Cell Migration Assay

A wound healing assay was used to evaluate cell migration. Briefly, cells were seeded and cultured to reach confluence, and then a wound was generated using a pipette tip. Mitomycin-C (5 mg/ml, purity: 99.74%, Selleck, S8146) was used to inhibit cell proliferation during this experiment. After an incubation of 0, 12, or 24 h, wound images were taken using a Model IX70 Microscope (Olympus, Tokyo, Japan). The width of wounds was measured and cell migration was calculated.

To observe the effect of metformin hydrochloride (MET, purity: 99.98%, MedChemExpress, HY-17471A) on cell migration, cells were pretreated with MET (100 μM) for 72 h, followed by cell migration analysis using wound healing assay as described above. The half-life of MET is 4–8.7 h (Dunn and Peters, 1995) and the media (containing glucose with or without MET) in this and other MET related experiments were changed every 24 h (Niu et al., 2019).

### *In Vitro* Angiogenesis (Tube Formation) Assay

The *in vitro* tube formation assay was performed in a 24-well plate using Growth Factor-Reduced Matrigel (Corning, 354234). Briefly, HUVECs were stained with cell-permeable dye, calcein (Corning, 354216), for 30 min and replanted in 24-well plates precoated with 150 μl/well growth factor-reduced Matrigel (Corning, 354234) and in cultured with ML385 (purity: 99.55%, MedChemExpress, HY-100523), TBHQ (purity > 98.0%, MedChem Express, HY-100489), Bay11-7082 (Bay, purity: 99.69%, Selleck, S2913) at 37°C in cell culture incubator. After a 12-h incubation, the formed capillary-like tubes were photographed in randomly chosen fields using a microscope (EVOS FL Imaging System, Thermo Fisher Scientific, Waltham, MA, USA). The tube formation was quantified by manual counting using ImageJ software (National Institutes of Health, Bethesda, Maryland, USA).

### The Enzyme-Linked Immunosorbent Assay (ELISA)

The serum samples were harvested from normal and GDM pregnant women, and the p65 and Nrf2 levels in serum were determined using the corresponding ELISA kits (Shanghai Wes Tang Bio-Tech Co., LTD, F02033, F02058, F04103, and F02309) according to the manufacturer’s protocols. The absorbance was measured using a microplate spectrometer (Thermo, USA).

### Western Blot

The protein samples were separated by SDS-PAGE and transferred to polyvinylidene fluoride membrane, followed by blockage with 5% bovine serum albumin (Sigma, B2064) in Tris-buffered saline (Sigma, T5030) containing 0.1% Tween 20 (Sigma, 93773) (TBST). The membranes were then incubated with primary antibodies at 4°C overnight. The primary antibodies included anti-NF-κB p65 (Abcam, ab16502), anti-Nrf2 (Abcam, ab31163), anti-GAPDH (glycer-aldehyde-3-phosphate dehydrogenase) (Abcam, ab9485), and anti-Lamin B1 (Proteintech, 66095-1-Ig) antibodies. After three washes with TBST, the membranes were incubated with HRP-goat-anti-rabbit (Abcam, ab6721) or HRP-goat-anti-mouse (Abcam, ab6789) secondary antibodies at room temperature for 1 h, followed by visualization using an enhanced chemiluminescence (ECL) system (Thermo Scientific, 32132). The images of protein bands were photographed using a ChemiDoc MP device (Bio-Rad, Hercules, CA, USA) and analyzed using ImageJ software.

### Extraction of Nuclear Protein

Cells were collected and lysed to obtain the nucleus and the cytosol lysates using Nuclear Protein and Cytoplasmic Protein Extraction Kit (Beyotime, P0028) according to the manufacturer’s protocols.

### Small Interfering RNA (siRNA) Experiment

The cells were seeded and reached a density of 50%–60% confluence. Then the cells in OptiMEM™ reduced serum medium (Gibco, 31985062) were transfected with the siRNA targeting the human *Nrf2* (Santa Cruz Biotechnology, Inc., sc-37030) and *p65* (Santa Cruz Biotechnology, Inc., sc-29410) using Lipofectamine 2000 (Invitrogen, L3000008). Twenty-four h after transfection, the cells were employed in *in vitro* angiogenesis assay to assess their angiogenesis function and in Western blot assay to detect the expression levels of related proteins.

### Analysis of Transcription Factor and the Binding Site of p65

The GDM gene expression data were acquired from the GEO database (accession number: GSE87295). Briefly, the transcriptomic profiles of 104 HUVEC samples (77 from GDM patients and 27 from normal control) were used for transcription factor prediction. The websites analytic system Jaspar (http://jaspar.genereg.net/) and PROMO (http://alggen.lsi.upc.es) were utilized to forecast the binding sites between p65 and *Nrf2* DNA.

### Luciferase Assays

293T cells were transfected with pGL4.10-NFE2L2 promoter (or truncated *NEF2L2* promotors), pcDNA3.1(+)-RELA, or pRL-CMV. After 24 h, the cells were plated in a 24-well plate (1 × 10^6^ cells/well) and cultured to reach confluence. The luciferase activity in cell lysates was then measured using a Dual-Luciferase Reporter Assay System (Spark 10M, TECAN) by following the manufacturer’s instructions. The Firefly luciferase and Renilla luciferase were measured using a Veritas Microplate Luminometer (Promega, Madison, WI, USA), and the Firefly luciferase: Renilla luciferase ratio was calculated and normalized to the ratio in only pcDNA3.1(+)- or pGL4.10(+)- transfected cells.

### Statistical Analysis

The data are shown as the mean ± standard error of the mean (SEM). GraphPad Prism version 5 was used for statistical analysis. Statistical significance was distinguished by the Student’s *t*-test for two groups, one-way analysis for multiple groups.

## Results

### MET Improves GDM-Attenuated Migration and Angiogenesis of HUVECs

**Figure 1:**
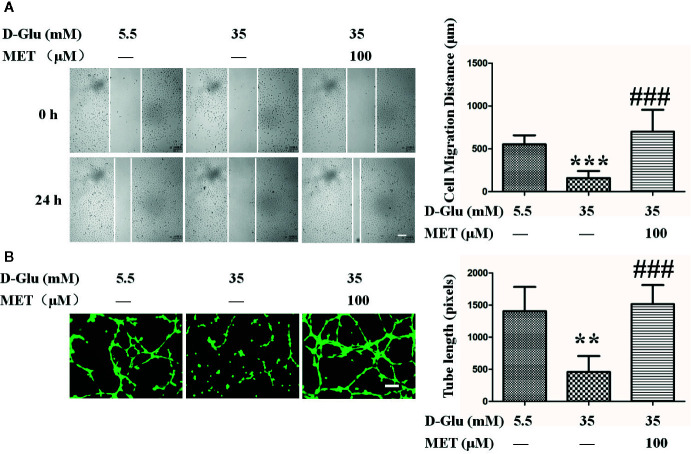
Metformin (MET) recovers gestational diabetes mellitus (GDM)-impaired the migration and angiogenesis of HUVECs. **(A)** A scratch wound assay was performed in the presence of 5.5 or 35 mM D-Glucose with or without MET (100 μM) for 72 h. Cell monolayers were imaged at 0, 24, 48, and 72 h after wounding. White vertical lines indicate the wound area borders. Space bar=500 μm. The images shown here were captured at 0 and 24 h. The cell migration distance was measured and shown on the right. **(B)** Tube formation assay was performed to assess the angiogenesis ability of HUVECs in the presence of 5.5 or 35 mM D-Glucose with or without MET for 72 h. Bars=50 mm. The tube length was quantified and shown on the right. All values displayed are mean ± SEM of 8 independent experiments. **P < 0.01, ***P < 0.001 vs. 5.5 mM D-Glucose group; ^###^P < 0.001 vs. 35 mM D-Glucose group.

To assess the protective effect of MET against GDM-induced endothelial dysfunction *in vitro*, the GDM cell model was constructed by exposing HUVECs to high glucose with or without MET. Above all, we found the normal postprandial blood glucose level should not exceed 11.1 mM in general, and previous studies used 33 or 30 mM glucose to treat cells to simulate gestation diabetes *in vitro* (Niu et al., 2019; Liu et al., 2020). In our study, the effects of different concentrations of glucose (5.5, 15, 35, and 55 mM) on cell migration and proliferation were observed (**Figures S1A**, **B**, **E**), and the results showed that 35 mM glucose exhibited the best cellular effects *in vitro*, therefore, 35 mM glucose (HG, 35 mM D-glucose) was used in our following cellular experiments. Comparing with normal group (NG, HUVECs were cultured in medium containing 5.5 mM D-glucose), we observed that HG significantly suppressed the cell migration, while MET recovered this inhibitory effect induced by HG (**Figure 1A**).

Moreover, the cells were treated with 5.5 or 35 mM glucose in the presence or absence of MET (25, 50, 100, or 200 μM) for 72 h. Results showed that 100 μM MET is the optimal concentration for inhibition of cell migration and proliferation (**Figures S1C**, **D**, **F**), therefore, 100 μM MET was used in the following experiments. In addition, tube formation results showed that the tube-forming activity of HUVECs was significantly suppressed by HG as compared to HUVECs maintained in NG, but this suppression was dramatically recovered by co-treatment with MET, as demonstrated by the measurement of tube length (**Figure 1B**).

### The Role of Nrf2 in Tube Formation Under the GDM (HG) Condition

**Figure 2:**
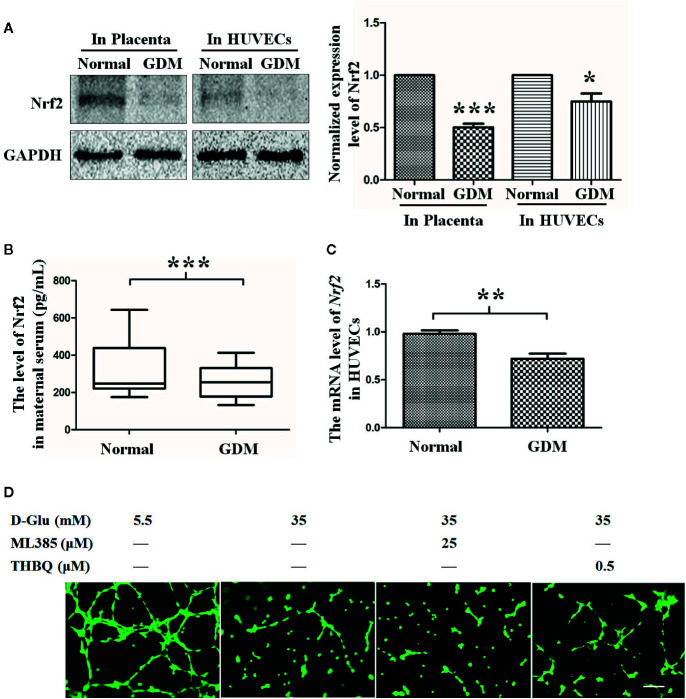
The role of Nrf2 in tube formation under the gestational diabetes mellitus (GDM) (HG) condition. **(A)** Western blot analysis of the Nrf2 level in normal and GDM placenta tissues and HUVECs. The Nrf2 protein levels were quantified and shown on the right (n=5). **(B)** Quantification of Nrf2 expression level in peripheral blood from normal and GDM pregnant women using Enzyme-Linked Immunosorbent Assay (ELISA). **(C)** Nrf2 expression level in normal and GDM HUVECs. (n=5). **(D)** Tube formation assay was performed to assess the angiogenesis ability of HUVECs in the presence of 5.5 or 35 mM D-Glucose with or without ML385 or THBQ for 72 h. HUVECs were cultured either in normal glucose (5.5 mM) or high glucose (35 mM) medium in the presence or absence of MET (100 μM) for 72 h. Bars=50 mm. All values displayed are means ± SEM of 8 independent experiments. *P < 0.05, **P < 0.01, ***P < 0.001 vs. 5.5 mM.

Numerous studies have shown that Nrf2 may be a key molecule through which risk factors elicit oxidative and inflammatory response to induce angiogenic disorders (Yan et al., 2019). In our study, we found the Nrf2 level was decreased in GDM placenta tissues (**Figure 2A**). Consistently, we also found the Nrf2 circulatory level was decreased in GDM mothers (**Figure 2B**). We also examined the Nrf2 level in primary HUVECs extracted and isolated from endothelial tissues of umbilical cord vein with or without GDM, and a lower Nrf2 level was observed in GDM HUVECs when compared with normal HUVECs at both protein and mRNA levels (**Figures 2A, C**).

To further explore the effect of Nrf2 on hyperglycemia-induced impairment of endothelial function, we observed the effect of upregulated Nrf2 on HG-induced endothelial function impairment. HG-exposed HUVECs were co-cultured with Nrf2 stimulator THBQ or inhibitor ML385. Similar to the results obtained under MET stimulation, coincubation with THBQ, to a large extent, alleviated HG-impaired angiogenesis, while ML385 had no significant effect on angiogenesis (**Figure 2D**). These results suggest that Nrf2 may play an important role in protecting endothelial function against HG.

### The Nrf2 Activation Regulated by MET

**Figure 3:**
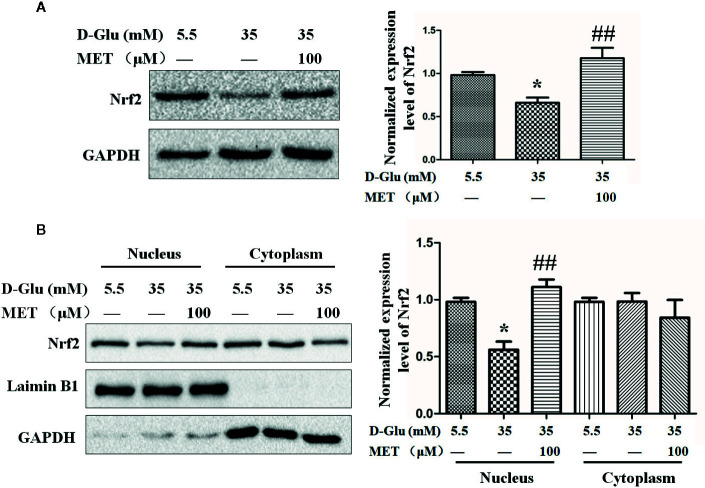
The upregulation of Nrf2 by metformin (MET). **(A)** Western blot analysis of the Nrf2 level in HUVECs treated with 5.5 or 35 mM D-Glucose with or without MET (100 μM) for 72 h. The Nrf2 level was quantified and shown on the right. **(B)** Western blot analysis of the Nrf2 level in nucleus and cytoplasm of HUVECs treated with 5.5 or 35 mM D-Glucose with or without MET (100 μM) for 72 h. The Nrf2 level was quantified and shown on the right. All the values displayed are mean ± SEM of 8 independent experiments. *P < 0.05 vs. 5.5 mM D-Glucose group; ^##^P < 0.001 vs. 35 mM D-Glucose group.

Based on the effects of MET and Nrf2 on tube formation, we next explored the relationship between MET and Nrf2. Following HG-induced downregulation of Nrf2, MET enhanced the expression level of Nrf2 (**Figure 3A**). Meanwhile, by analyzing the separated cytoplasmic and nuclear proteins, we found MET increased the HG-reduced Nrf2 accumulation in nucleus, but not in cytoplasm (**Figure 3B**). In addition, the protein level of heme oxygenase (HO-1), which is regulated by Nrf2, was also examined, and the results showed that MET could also increase the protein level of HO-1 (**Figures S2A**, **B**). Considering the antioxidative function of Nrf2, we tested the influence of MET on the oxidative stress. ELISA results showed that HG increased the levels of 8-isoprostanes and carbonyls, the biomarkers of oxidative stress, while MET attenuated the oxidative stress induced by HG (**Figures S2C, D**).

### Nrf2 Expression May Be Regulated by p65 at Transcriptional Level

**Figure 4:**
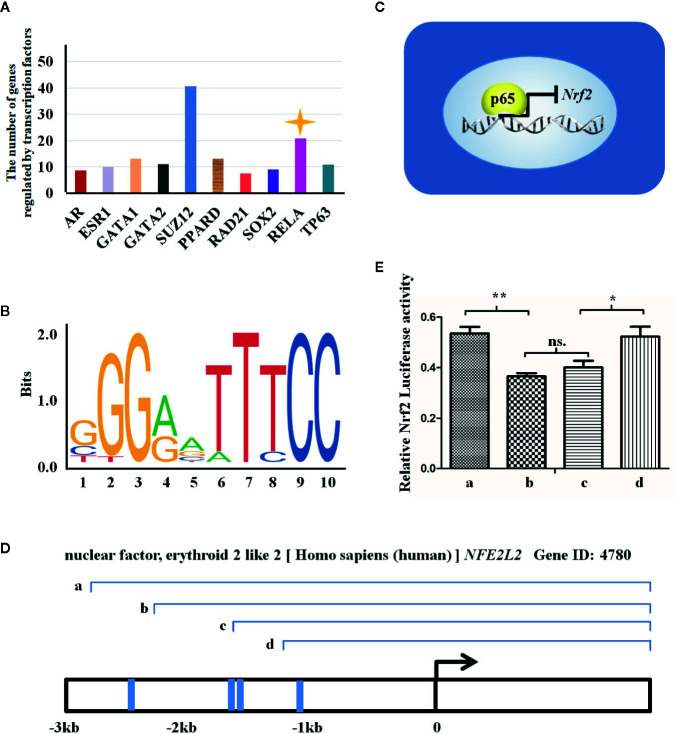
Nrf2 expression may be regulated by p65 at transcriptional level. **(A)** There are transcription factors regulating the expression of genes induced by gestational diabetes mellitus (GDM), which are revealed in the X axis. The Y axis indicates the number of genes regulated by the transcription factors. **(B)** The figure reveals the sequence logo of p65 from JASPAR Web (http://jaspar.genereg.net/matrix/MA0107.1/). **(C)** The figure reveals the way by which p65 regulates the transcription of *NFE2L2* in GDM HUVECs. **(D)** The transcriptional mechanism of p65 and the three transcriptional binding sites for p65 in *NEF2L2* promoter region. Blue line representes the locations of binding sites, and a, b, c, and d represent the potential sites regulated by p65. **(E)** Luciferase reporter gene assay employed to assess which sites are transcriptional binding sites for p65 in Nrf2 promoter region. a, b, c, and d represent the truncated promoter subsequence indicated in **(C)**. All data are presented as mean ± SEM of 5 replicates, *P < 0.05, **P < 0.01 compared with the indicated group.

Increasing evidence have shown that MET exerts its anti-oxidative and anti-inflammatory activities *via* suppression of NF-κB while activation of Nrf2 signaling pathways (Chiang et al., 2017; Ye et al., 2018). We next wan to elucidate whether NF-κB and Nrf2 signaling pathways are independent or crosstalk with each other under the GDM condition. The RNA-sequencing data (named as GSE87295) analyzing GDM and normal HUVECs obtained from GEO database was used to predict the transcriptional factors which regulate expression of different genes. The bioinformatics analysis uncovered that the number of differentially expressed gene (DEG) was controlled by transcriptional factors, among which p65 (RELA, one subunit of NF-κB dimer) ranked the second, modulating about 20 DEGs (**Figure 4A**). In virtue of JASPAR website, we also ascertained the acid bases in the p65 binding sequence (**Figure 4B**) and the four binding sites in the *Nrf2* promotor region (also known as *NFE2L2*) for p65 transcription binding, namely -1063/-1053, -1415/-1404, -1633/-1624, and -2672/-2601 (**Figures 4C, D**).

According to the above predicted results, we successively truncated the *Nrf2* promoter region (**Figure 4D**), and conducted luciferase assay on the successively truncated promoter. The results showed that p65 regulated *Nrf2* through transcription of four binding sequences in the *Nrf2* promoter region. Segment a (containing the above four promoter regions) promoted the transcription of *Nrf2* by p65, while segment b and c (containing the above three and two promoter regions, respectively) inhibited the transcription of *Nrf2* by p65, while segment d (containing the above one promoter region) promoted the transcription of *Nrf2* by p65 (**Figure 4E**).

These observations suggest that the evaluated expression level of *Nrf2* under MET treatment is mainly ascribed to the p65-transcriptional regulation.

### The Expression of p65 Is Elevated in GDM Peripheral Blood, Placenta, and HUVECs, and Suppression of p65 Could Recover GDM-Induced Suppression of Angiogenesis

**Figure 5:**
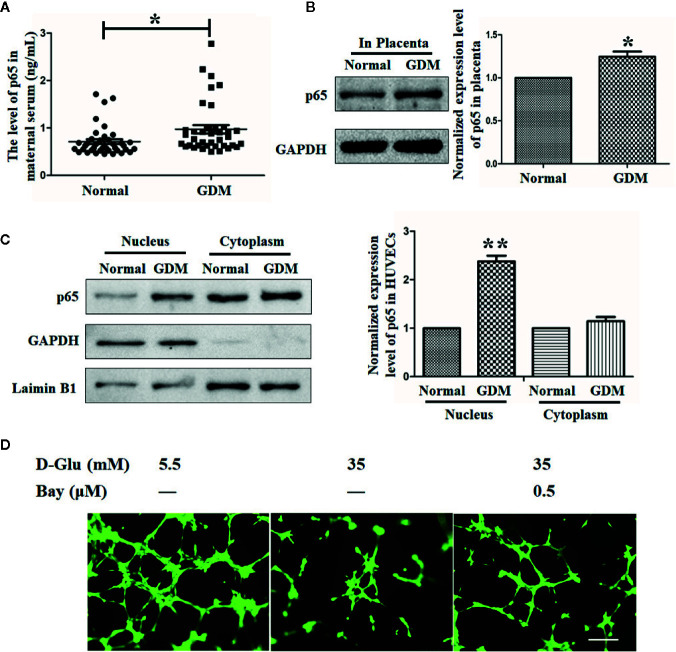
The expression of p65 is elevated in gestational diabetes mellitus (GDM) peripheral blood, placenta, and HUVECs, and suppression of p65 could recover GDM-induced suppression of angiogenesis. **(A)** Enzyme-Linked Immunosorbent Assay (ELISA) assay was performed to evaluate the level of p65 in normal and GDM maternal serum. **(B)** Western blot analysis of the p65 level in normal and GDM placenta tissues. **(C)** p65 expression level in normal and GDM HUVECs was determined using Western blot with extracted nuclear or cytoplasmic proteins. The p65 level was quantified and shown on the right. **(D)** Tube formation assay was performed to assess the angiogenesis ability of HUVECs treated with 5.5 mM or 35 mM D-Glucose with or without 0.5 μM Bay11-7082 (Bay) () for 72 h. Bars = 50 mm. All data are presented as mean ± SEM of 5 replicates, *P < 0.05, **P < 0.01 compared with the indicated group.

Substantial evidence has demonstrated the critical roles of p65 in the process of GDM, as well as in the related inflammation and angiogenesis processes (Kuzmicki et al., 2013; Cui et al., 2019). Consistent with the phenomena observed in previous studies, in our study, we observed an elevated p65 level in peripheral blood subjected to GDM (**Figure 5A**). By the same token, the p65 level in placenta tissue was also observed to be increased (**Figure 5B**).

By analyzing the separated cytoplasmic and nuclear proteins of primary HUVECs isolated from endothelial tissues of umbilical cord vein with or without GDM, we observed an increased p65 level in nucleus but not in cytoplasm under the GDM condition (**Figure 5C**).

Moreover, the tube formation results indicated that HG dramatically impaired the angiogenesis ability of HUVECs, while the reduction of p65 expression induced by Bay11-7082 under HG situation recovered the angiogenesis ability of HUVECs (**Figure 5D**), suggesting that the antiangiogenic effect of HG (or GDM) is mediated at least in part by activation of p65.

### MET Restores the GDM-Induced Angiogenesis Impairment May *Via* Downregulation of p65 and Upregulation of Nrf2

**Figure 6:**
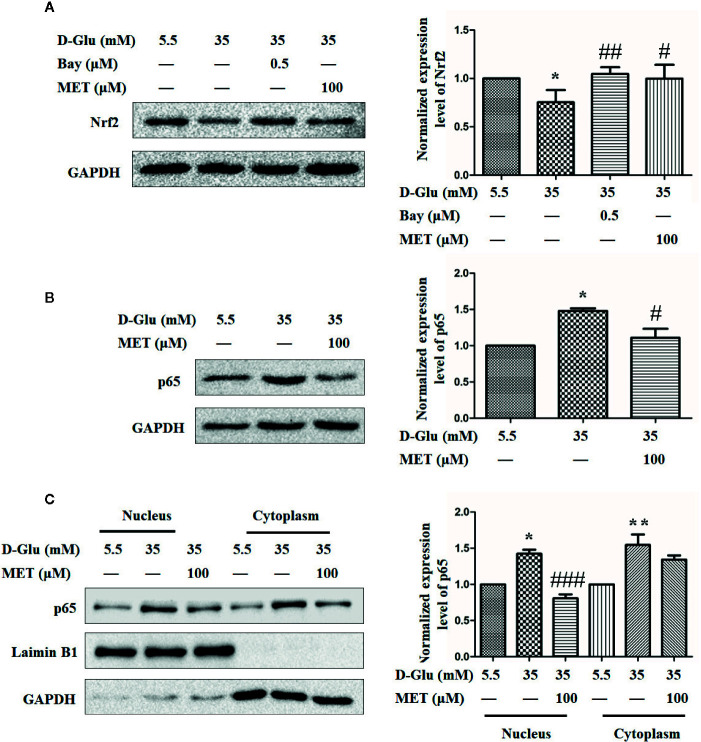
Metformin (MET) restores the gestational diabetes mellitus (GDM)-induced angiogenesis impairment *via* downregulation of p65 and upregulation of Nrf2. **(A)** Nrf2 expression level in HUVECs treated with 0.5 μM Bay11-7082 (Bay) or 100 μM MET determined using Western blot. The Nrf2 level was quantified and shown on the right. **(B)** Western blot analysis of the p65 expression level in HUVECs treated with 5.5 or 35 mM D-Glucose with or without 100 μM MET. The p65 level was quantified and shown on the right. **(C)** Western blot analysis of nuclear and cytoplasmic p65 level in HUVECs treated with 5.5 or 35 mM D-Glucose with or without MET. Preparation of nuclear and cytoplasmic lysates and western blot were performed as described in the Methods section. The p65 level was quantified and shown on the right. All data are presented as mean ± SEM of 5 replicates, *P < 0.05 compared with normal group; ^#^P < 0.05, ^##^P < 0.01, and ^###^P < 0.001 compared with 35 mM D-Glucose treatment group.

The mechanism underlying the protective effect resulted from MET-mediated Nrf2 activation against GDM-impaired angiogenesis alleviating was further explored. In the light of our previous results that *Nrf2* may be transcriptionally regulated by p65, we treated HUVECs with either Bay11-7082 or MET in the presence of 35 mM D-glucose. **Figure 6A** showed that HG induced significant downregulation of Nrf2, while both Bay11-7082 and MET can restore this downregulation of Nrf2 induced by HG. Similarly, both MET and Bay11-7082 enhanced the expression level of HO-1 even under HG condition (Fig. S2A and B). Moreover, we also observed that MET downregulated the level of p65 when stimulated by 35 mM D-glucose (**Figure 6B**). Based on this result, we also detected the levels of p65 in nucleus and cytoplasm, and the results revealed that MET decreased the accumulation of p65 in both nucleus and cytoplasm induced by HG (**Figure 6C**). These results suggest that MET restore GDM-impaired angiogenesis may *via* downregulation of p65 and upregulation of Nrf2.

## Discussion

Numerous studies have shown that Nrf2 plays a critical role in GDM, and the activation of Nrf2 can exert protective effect against GDM-induced oxidative stress (Manoharan et al., 2019). Although insulin has been seemed as the gold standard for hyperglycemia treatment during pregnancy (Park et al., 2015), in this study, we demonstrated that MET promoted angiogenesis against GDM-induced impairment *via* enhancing Nrf2 expression and downregulating transcriptional factor p65, which could provide a new theoretical basis for the therapy of gestational diabetes, not only *via* controlling blood glucose levels, but also *via* improving endothelial dysfunction.

Different studies have explored the effects of MET in HUVEC from different perspectives. MET exerted protective effect against hyperglycemia-induced endothelial impairment, which was contributed to MET improving HG-inhibited GLI1 activity and BNIP3 expression in HUVECs (Niu et al., 2019). MET was also reported to promote angiogenic potential of HUVECs probably by the regulation of endocan dynamics under high glucose condition (Zolali et al., 2019) and modulate high glucose-induced HUVECs proliferation and apoptosis through AMPK/CREB/BDNF Pathway (Han et al., 2018). However, our study focused on exploring the mechanism of MET in modulating hyperglycemic-induced oxidative stress in order to open a new perspective of MET in the treatment of GDM (HG). Anti-oxidant reaction is a process led by environmental stress including high glucose, however, the way of anti-oxidant reaction differs. Recent research has observed that activation and upregulation of Nrf2 could dramatically alleviate GDM-provoked oxidation, and Nrf2 has been identified as an important antioxidative regulator for activation of antioxidant defense genes and recovery of vascular redox homeostasis (Song et al., 2019). Moreover, Cheng et al. verified that Nrf2 overexpression in GDM endothelial cells could recover its target NAD(P)H:quinone oxidoreductase 1 (NQO1) gene. On the other hand, they demonstrated that Nrf2 dysregulation in fetal endothelium led to increased risk of cardiovascular disease and type 2 diabetes (Ishii et al., 2000). Our study demonstrated that MET administration significantly elevated HG-lowered Nrf2 expression level in HUVECs. Meanwhile, the HG-impaired angiogenesis was significantly restored or at least ameliorated by MET or Nrf2 activator THBQ, indicating a significant protective effect of MET and Nrf2 in HG-impaired antioxidative and angiogenesis abilities. Consistent with our results, applying GDM mice model, people demonstrated that oxidative stress occurred in pregnant GDM mice, triggering lower expression of nuclear Nrf2 and heme oxygenase-1 (HO-1) and downregulating the oxidative responsive ability (Chen et al., 2020). It is worth noting that our results in conjunction with other similar research results mainly focus on the effect of MET on HG-impaired anti-oxidation and angiogenesis, and more research focusing on the roles of Nrf2 and its downstream genes regulated by MET in endothelial function under HG conditions needs to be done in the future.

In this study, the role of Nrf2 in response to MET under HG condition was explore. Consistent with our results, MET has been previously reported to be able to regulate Nrf2, especially in the context of disease induced by oxidative injury (Liu et al., 2019). During the process of HG-caused oxidative stress and angiogenic impairment, Nrf2 has been identified as a potential target of MET for promoting angiogenesis under the HG situation. Substantial previous reports have demonstrated the antioxidative activity of MET *via* Nrf2 and its target genes (Yan et al., 2019; Jia et al., 2020), indicating that MET could exert its antioxidative effect, at least partially through Nrf2. In this study, we found that Nrf2 expression was suppressed by GDM/HG, and administration of MET could reversely increase the Nrf2 level and its nuclear stabilization under GDM/HG condition to enhance the angiogenic ability. These observations implies that Nrf2 is involved in MET-induced restoration of HG-impaired antioxidative and angiogenic capacities, although a causal role of MET in regulation of Nrf2 and downstream genes under GDM/HG circumstances needs further studies.

In the light of *Nrf2* level change in GDM condition, we further explored the cause of the Nrf2 level alteration. Previous reports showed that placentae of GDM mother patients and nascent nephron of hyperglycemic patients displayed a remarkable overexpression of p65 and relative inflammatory factors (Chen et al., 2011) and p65 has been recognized as a central molecule to regulate the expression of oxidative stress- and inflammatory-responsive genes in gestational tissues, including the placenta and umbilical cord blood (Barnes and Karin, 1997; Haddad, 2002; Lappas et al., 2002), indicative of the pivotal role of p65. In our study, bioinformatic analysis and luciferase assay showed that *Nrf2* was transcriptionally regulated by transcription factor p65 in virtue of four binding sites at *Nrf2* promotor region. Intriguingly, in acute myeloid leukemia cells high nuclear Nrf2 expression level was demonstrated to be regulated by high nuclear p65 level (Herpers et al., 2016), which is coincident with our assumption. However, the relationship between Nrf2 and p65 remains paradoxical. Bram Herpers et al. uncovered that Nrf2 activation is closely associated with suppression of NF-κB activity in primary human hepatocytes (Abd El-Twab et al., 2019). On the contrary, Antonio Cuadrado et al. reported the existence of a crosstalk between p65 and Nrf2 during the modulation of inflammation by a small GTPase protein RAC1 (Cuadrado et al., 2014). Therefore, the exact relationship between Nrf2 and p65 should be analyzed under certain circumstance.

**Figure 7:**
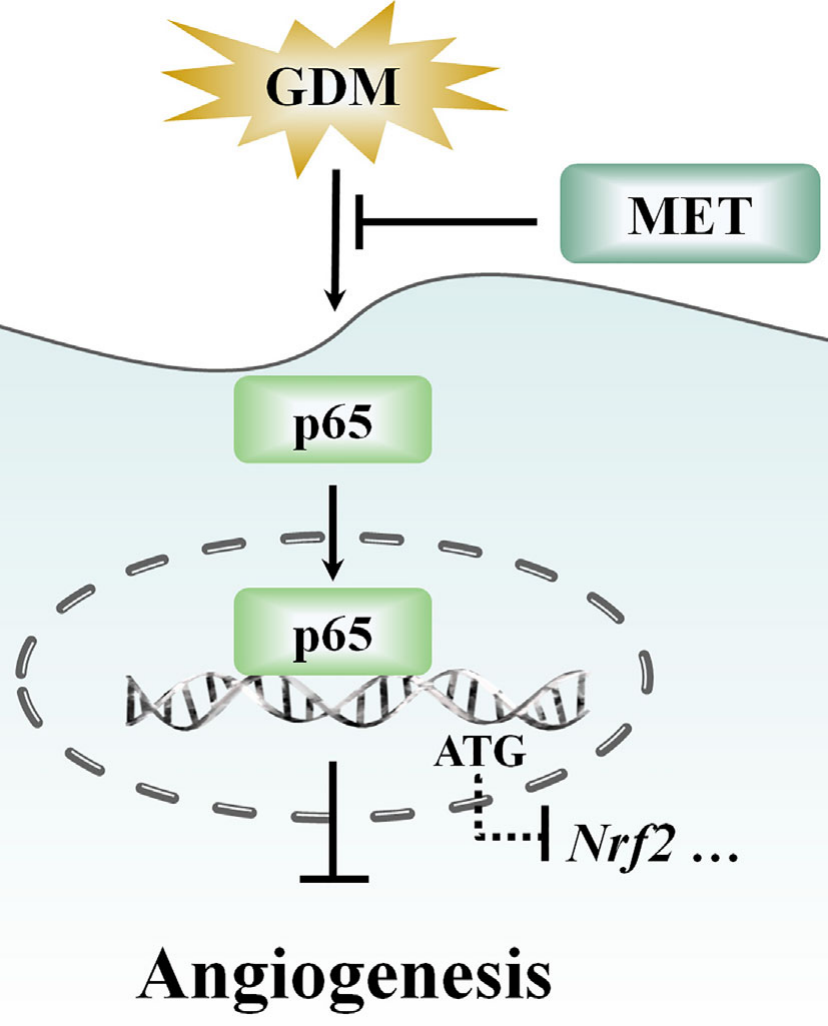
Schematic showing that metformin (MET) alleviates hyperglycemia-induced impairment of endothelial angiogenesis *via* p65-*Nrf2* pathway. MET treatment suppresses the gestational diabetes mellitus (GDM)-induced p65 upregulation and activation, and thereby altering the p65 transcription level through the translocation of p65 into nucleus. Moreover, MET also increases GDM-induced downregulation of Nrf2 expression.

Furthermore, with the progressive shift from a “cure” to “prevention” focus (Miquel et al., 2018; Leri et al., 2020), more and more drug research has focused on prevention of diseases and exploration of new functions, such as polyphenols, myopeptides, and GABA-containing compound gammapyrone (Peters et al., 2018; Pilipenko et al., 2019; Brunetti et al., 2020; Di Rosa et al., 2020). In recent years, in addition to glucose lowering effect, several studies have presented evidence showing some potential roles for MET, such as antitumor effect, antiaging effect, cardiovascular protective effect, neuroprotective effect, or an optional treatment for polycystic ovary syndrome (Wang et al., 2017). We have demonstrated that MET plays an important role in remitting oxidative stress and inflammation (**Figure 7**), and advancing endothelial function in GDM pregnancy women. Given the relationship between inflammation and redox status network, and its potential biological functions in neuroprotection and metabolism (Calabrese et al., 2010), the efficacy of MET on other diseases, including neuroprotection should not be neglected, which is beneficial to exploring other potential therapeutic functions of MET.

Moreover, our results showed that different concentrations of MET had different effects on cell proliferation and migration (SF. 1), suggesting hormesis in involved in cellular protection. Consistent with our inference, more research revealed that hormesis is related to endogenous cellular defense pathways, including Nrf2 and related pathways that integrate adaptive stress responses (Calabrese et al., 2018a; Calabrese et al., 2018b; Calabrese et al., 2020). It was reported that similar to MET, polyphenols, extracted from olive leaves and drupes can act as stressors to activate various cell defense systems, controlling redox environment, metabolic homeostasis, organelle turnover, and inflammation (Calabrese et al., 2010). Further, olive oil polyphenols also been demonstrated to reduce insulin resistance and to improve impaired glucose homeostasis (Leri et al., 2020; Di Rosa et al., 2020), however, there are few reports about the difference in cell protection mechanism of polyphenols and MET, which needs further study. Understanding this mechanism could improve the usage accuracy of these pharmaceutical agents within the highly heterogeneous human population.

Collectively, our study demonstrated the protective effect of MET on GDM/HG-induced impairment of angiogenesis capability *via* Nrf2. Moreover, we also laid the groundwork for assumption that Nrf2 may be transcriptionally regulated by p65 *via* the four predicted binding sites at *Nrf2* promotor region. These results revealed a new mechanism for MET regulation of GDM-triggered endothelial dysfunction.

## Data Availability Statement

The raw data supporting the conclusions of this article will be made available by the authors, without undue reservation.

## Ethics Statement

The studies involving human participants were reviewed and approved by Ethics Committee of Wenzhou People’s Hospital. The patients/participants provided their written informed consent to participate in this study.

## Author Contributions

CCS and YNL conducted the experiments, performed data analysis, and wrote the paper, participated in the experimental process together. WHW, XMX, XQL, and HW supported the study and critically revised the paper. JYZ and JQZ conceived and designed the study. All authors contributed to the article and approved the submitted version.

## Funding

This work was supported by grants from the Natural Science Foundation of Zhejiang Province (LQ18H040002 and LY19H040008), Zhejiang Medical and Health Science and Technology Project (2018RC064), the Pharmaceutical Association Hospital pharmacy special research funding project of Zhejiang (2018ZYY45), the medical and health research project of Wenzhou (2018B03), and the Fundamental Scientiﬁc Research Project of Wenzhou (Y20180020 and Y20190047).

## Conflict of Interest

The authors declare that the research was conducted in the absence of any commercial or financial relationships that could be construed as a potential conflict of interest.

## Abbreviations

EBM, endothelial cell basal medium; EPCs, endothelial progenitor cells; GDM, gestational diabetes mellitus; HO-1, heme oxygenase-1; MET, metformin; NQO1, NAD(P)H,quinone oxidoreductase 1; Nrf2, nuclear factor erythroid 2-related factor 2; OGTT, oral glucose tolerance test; siRNA, small interfering RNA; T2DM, type 2 diabetes mellitus; VECs, vascular endothelial cell.
