# Supplementary material for: Metformin Ameliorates Gestational Diabetes Mellitus-Induced Endothelial Dysfunction via Downregulation of p65 and Upregulation of Nrf2
Source: Front Pharmacol. 2020 Oct 9;11:575390. doi: 10.3389/fphar.2020.575390 (PMC7581851; doi:10.3389/fphar.2020.575390)
Supplement: Supplementary file 1 [file Presentation_1.pdf]

Material and methods

CCK-8 assay

Cell survival rates were estimated by the CCK-8 assay (MedChemExpress, HY-K0301). Approximately 5×10^4 cells were seeded in 96-well plates with 100 μL medium each well. After 24 h cultivation, different doses of glucose and/or MET were added respectively and incubated for 72 h. Each well was incubated with 10 μL CCK-8 solution for 2 h away from light before measuring the absorbance at 450 nm by Microplate Reader (Molecular Devices, Flexstation® 3).

Figure legends

Fig. S1 Effects of different concentrations of sugar and metformin on cell proliferation and migration. (A, B, C, D) A scratch wound assay was performed in the presence of 5.5, 15, 35 or 55 μM glucose (A) and 35 mM glucose with/without 25, 50, 100 or 200 μM MET (C) treatment for 72 h. Cell monolayers were imaged at 0, 24, and 48 after wounding. White vertical lines indicate the wound area borders. Space bar=100 μm. The images shown here were captured at 0 and 24 h. The cell migration distance was measured and shown in (B) and (D) respectively. (E, F) CCK8 assay was used to detect the chemo-sensitiviy of 5.5, 15, 35 or 55 μM glucose (E) and 35 mM glucose with/without 25, 50, 100 or 200 μM MET (F) treatment. The survival rate = (mean absorbance of experimental group/mean absorbance of control group) × 100%. All the values displayed are mean ± SEM of 5 independent experiments. *P< 0.05, ***P< 0.001 vs. 5.5 mM D-Glucose group; #P < 0.05, ##P < 0.01, ###P < 0.001 vs. 35 mM D-Glucose group.

Fig. S2 The effect of MET on oxidative stress under high glucose treatment in HUVECs. (A, B) Western blot analysis of the HO-1 level in HUVECs treated with 5.5 or 35 mM D-Glucose with or without MET (100 μM) or Bay11-7082 (0.5 μM) for 72 h. The HO-1 level was quantified and shown in (B). (C, D) ELISA assay was performed to evaluate the level of protein carbonyl (C) and 8-isoprostane (D) in HUVECs treated with 35 mM D-Glucose with or without MET (100 μM). All the values displayed are mean ± SEM of 5 independent experiments. ***P< 0.001 vs. 5.5 mM D-Glucose group;

#$P < 0.05$, ##$P < 0.01$, ###$P < 0.001$ vs. 35 mM D-Glucose group.
