# Supplementary figures and images for: Metformin Ameliorates Gestational Diabetes Mellitus-Induced Endothelial Dysfunction via Downregulation of p65 and Upregulation of Nrf2
Source: Front Pharmacol. 2020 Oct 9;11:575390. doi: 10.3389/fphar.2020.575390 (PMC7581851; doi:10.3389/fphar.2020.575390)

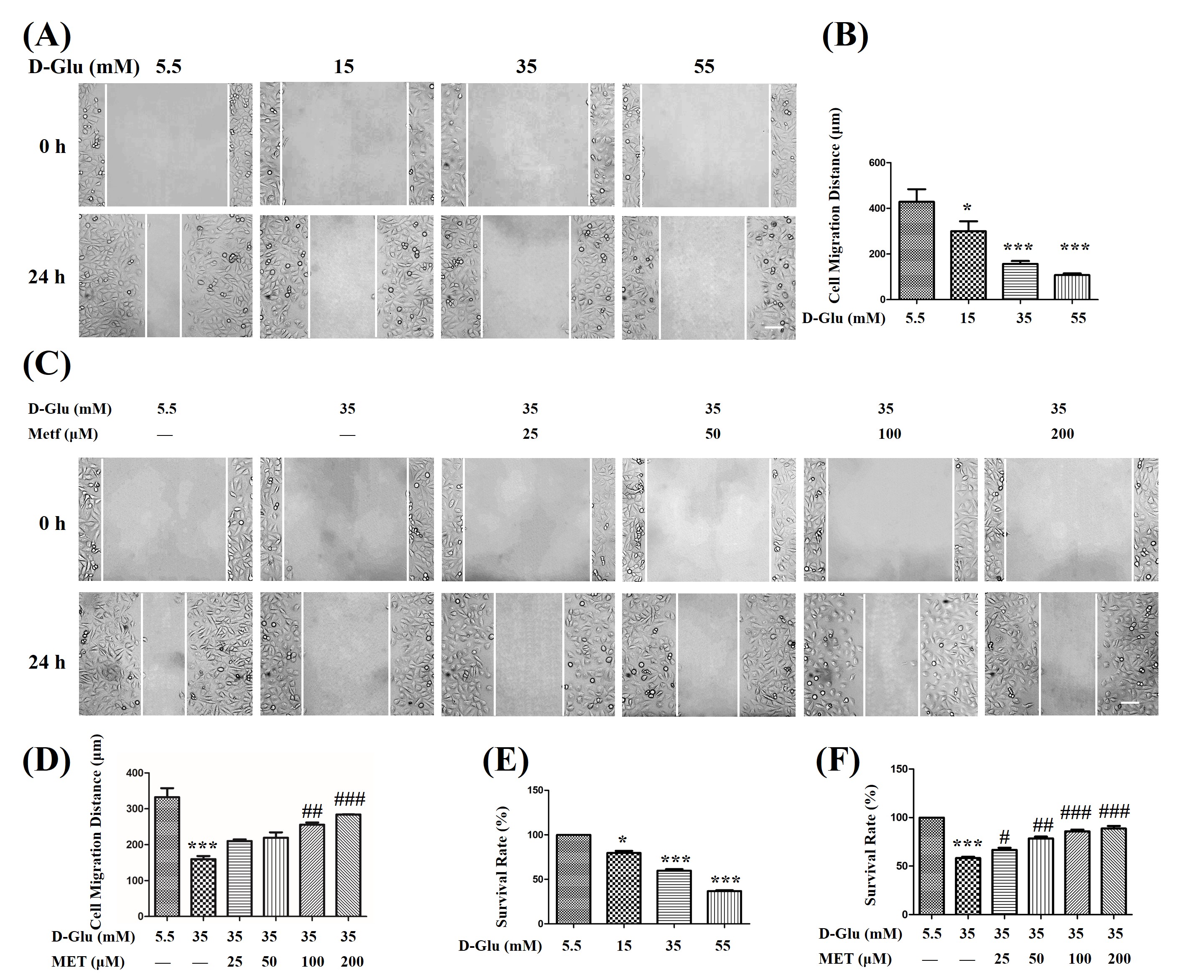

Supplement: Supplementary file 2 [file Image_1.jpg]

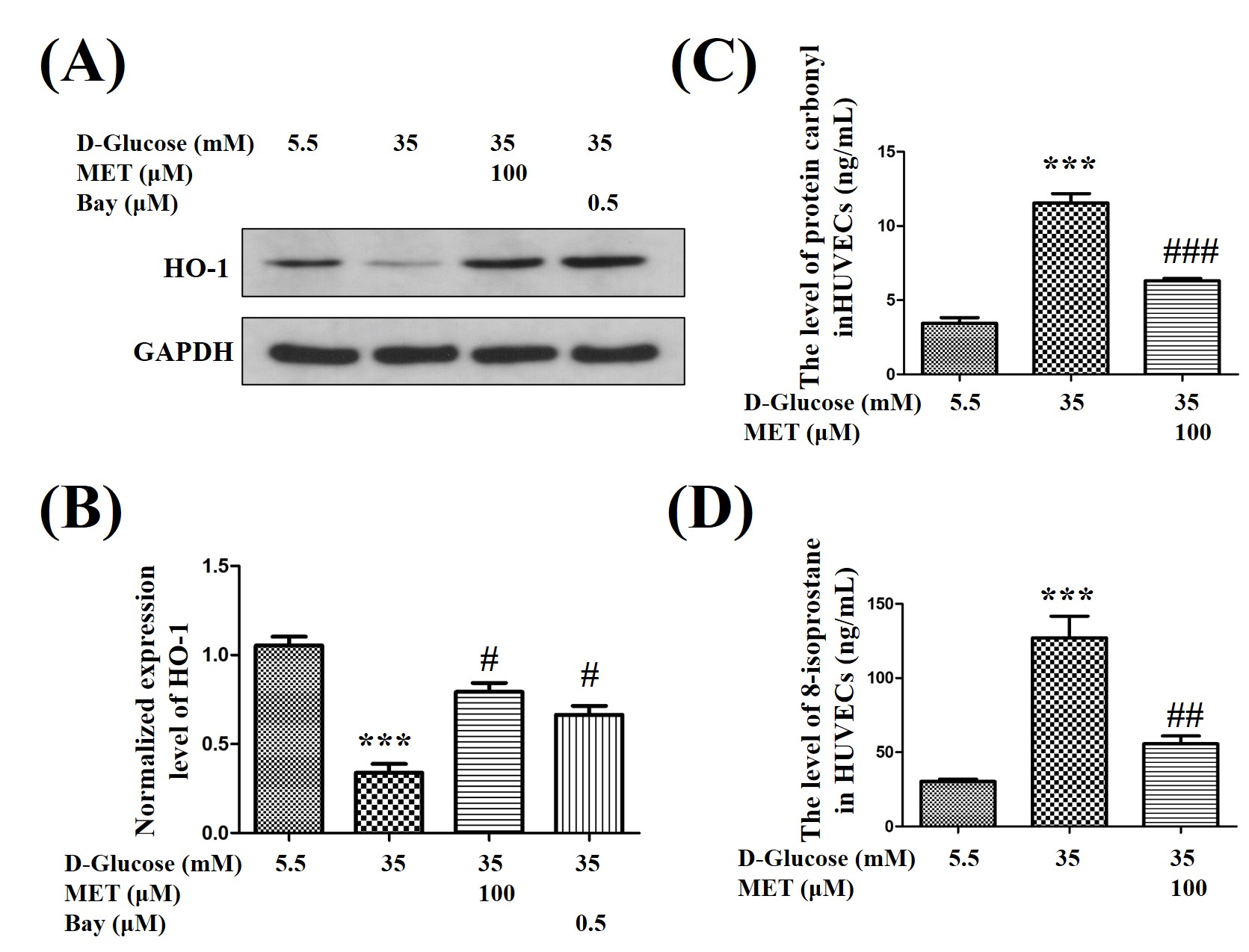

Supplement: Supplementary file 3 [file Image_2.jpg]
